# Mannose-decorated ginsenoside Rb1 albumin nanoparticles for targeted anti-inflammatory therapy

**DOI:** 10.3389/fbioe.2022.962380

**Published:** 2022-08-15

**Authors:** Zhihui Fu, Xiaohui Wang, Xuan Lu, Ying Yang, Lingling Zhao, Lin Zhou, Kaikai Wang, Hanlin Fu

**Affiliations:** ^1^ The First Affiliated Hospital of Zhengzhou University, Zhengzhou, China; ^2^ School of Pharmacy and Affiliated Hospital of Nantong University, Nantong University, Nantong, China

**Keywords:** ginsenoside, mannose, albumin nanoparticles, targeted delivery, inflammation

## Abstract

Ginsenoside Rb1 is a potential anti-inflammatory natural molecule, but its therapeutic efficacy was tremendously hampered by the low solubility and non-targeted delivery. In this study, we innovatively developed a mannose (Man)-modified albumin bovine serum albumin carrier (Man-BSA) to overcome the previously mentioned dilemmas of Rb1. The constructed Man-BSA@Rb1 NPs could improve the solubility and increase the cellular uptake of Rb1, finally leading to the enhanced anti-inflammatory effects. The robust therapeutics of Man-BSA@Rb1 NPs were measured in terms of nitrite, tumor necrosis factor-α (TNF-α), and interleukin-6 (IL-6) levels, which might be achieved by potently inhibiting nuclear factor-κB (NF-κB) and mitogen-activated protein kinase (MAPK) signaling pathways in lipopolysaccharide (LPS)-induced Raw264.7 cells. Moreover, the therapeutic efficacy of Man-BSA@Rb1 NPs was further confirmed in the d-Gal/LPS-induced liver injury model. The results indicated that Man-BSA may offer a promising system to improve the anti-inflammatory therapy of Rb1.

## 1 Introduction

Inflammation presents a potential threat to human health, and the unbalanced release of some inflammatory mediators will lead to the occurrence of related diseases such as sepsis shock, arthritis, and inflammatory bowel disease (IBD) (Zhou et al., 2019; Wang Q. et al., 2020; Wang Z. et al., 2020). To date, a variety of herbal-based therapeutic approaches have been attempted to attenuate inflammatory responses. Among them, ginsenoside Rb1 extracted from ginseng has been extensively explored due to its biological functions, including anti-inflammatory, anti-oxidative stress, anti-apoptosis, and acceptable biosafety (Zhou and Xie, 2019; Zhang X. et al., 2021; Jiang et al., 2021). However, the low water solubility and poor bioavailability limit its further application.

A strategy that potentially overcomes the dilemma described above is to utilize nanoparticle (NP)-based materials as delivery carriers (Su et al., 2018; Mitchell et al., 2021; Zhao et al., 2021). For example, polyethylene glycol (PEG) and poly lactic-co-glycolic acid (PLGA) applied Rb1 NPs were generated to enhance the bioavailability of Rb1 (Zhou et al., 2021). Trimethyl chitosan derivatives developed Rb1 NPs that were generated to improve the oral absorption of Rb1 (Chen et al., 2021). Platelets constructed biomimetic Rb1 NPs that were prepared to improve the bioavailability and stability of Rb1 (Yin et al., 2022). Despite all the progress made by far, the off-target effect remains a bottleneck that limits the therapeutic index of Rb1 NPs. Therefore, it is necessary to develop new Rb1 NPs which contain satisfying properties of biocompatibility, safety, and targeting ability.

The fate of fabricated NPs usually depends on their constituent materials, including various organic or inorganic materials, followed by forming organic NPs (e.g., liposomes, micelles, and polymeric nanoparticles) or inorganic NPs (e.g., magnetic and carbon NPs). With the help of developed nanotechnology, versatile NPs have been extensively explored to offer improved properties of loaded cargos, such as drug loading and therapeutic efficiency (Li and Wang, 2020; Wu et al., 2020; Li Z. et al., 2021). However, NP-associated toxicity and immunogenicity remain as a challenge. Compared with the reported carriers, bovine serum albumin (BSA) is attractive to deliver low solubility and poor bioavailability drugs because it is biodegradable, biologically compatible, and possesses multiple drug-binding abilities (Sleep, 2015; Mazzaferro and Edwards, 2020). Encouragingly, BSA-bound paclitaxel (Abraxane) NPs have been used in clinical treatment of breast cancer (Hama and Ishima, 2021). To endow BSA with targeting ability, mannose (Man) ligand is introduced to target mannose receptors that are overexpressed in pro-inflammatory cells, such as macrophages (Chen and Huang, 2020; Jaynes and Sable, 2020). Man, a simple sugar, has been used in the field of dietary supplements. The advantages of the Man-based ligand are reflected in nontoxicity, biodegradation, and no immunogenicity. Importantly, mannose treatment suppresses macrophage activation through impairing IL-1β production (Torretta et al., 2020; Wang et al., 2021). Thus, the carrier of Man-BSA can deliver the drug into specific cells by the receptor-mediated endocytosis pathway, resulting in enhanced cellular uptake and inflammation alleviation. Moreover, compared with launched NPs (e.g., liposomes), Man-BSA-based NPs can not only protect the degradation of drug in the blood but also promote drug release under acidic conditions (Zhang et al., 2015; Zhu et al., 2015; Lei et al., 2021).

**SCHEME 1:**
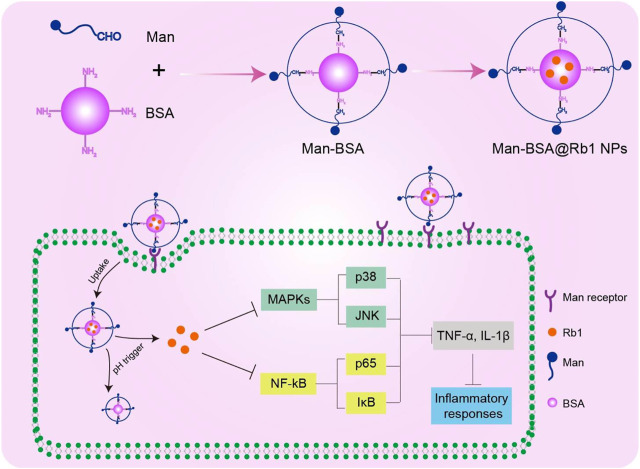
Schematic illustration of the preparation and mechanism of Man-BSA@Rb1 NPs.

In the present study, we report on the synthesis of Man-BSA and then prepared Man-BSA@Rb1 NPs with BSA@Rb1 NPs as controls. As shown in Scheme 1, Man-BSA@Rb1 NPs could improve Rb1 accumulation into the cells through mannose receptor targeted delivery. After endocytosis, Rb1 could be released in a pH trigger mode to play an anti-inflammatory response by inhibiting nuclear factor-κB (NF-κB) and mitogen-activated protein kinase (MAPK) signaling pathways. The physicochemical characterization of NPs was examined, including structure verification, morphology, particle size, zeta potential, stability, and drug loading and release. Furthermore, the anti-inflammatory effect of Man-BSA@Rb1 NPs was verified in lipopolysaccharide (LPS)-stimulated Raw264.7 cells *in vitro* and d-Gal/LPS-induced liver inflammation model *in vivo*.

## 2 Materials and methods

### 2.1 Chemicals and reagents

The Reactive Oxygen Species (ROS) Assay Kit (cat.no. S0033S), Fluo-4 AM (cat.no S1060), and Nitrite Assay Kit (cat.no S0023) were purchased from Beyotime Biotechnology (Shanghai, China). d-Mannose, BSA, and sodium cyanoborohydride were purchased from Aladdin Bio-Chem Technology Co., Ltd. (Shanghai, China). LPS and d-galactosamine (d-Gal) were purchased from Sigma-Aldrich (United States ). The antibodies of p-p65 (3033s), p65 (8242s), p-IκB-α (4812), IκB-α (2859), p-p38 (4511s), p38 (8690s), and β-actin (3700) were purchased from Cell Signaling Technology (United States ). The antibodies of p-JNK (76572) and JNK (179461) were purchased from Abcam Co., Ltd. (USK). All other reagents were obtained from Nanjing Jiancheng Bioengineering Institute (Shanghai, China) unless otherwise stated.

### 2.2 Synthesis of Man-BSA

Man-BSA was synthesized and purified according to a reductive amination method. In brief, d-mannose (100 mg), BSA (68 mg), and sodium cyanoborohydride (100 mg) were co-dissolved in 5.0 ml borate buffer (pH 8.0), and the reacted solution was incubated at 37°C for 24 h. The reaction was terminated by adjusting the pH of the system to 4.0 with acetic acid. The final product of Man-BSA was purified by dialyzing against deionized water three times (molecular weight cut off 1.0 kDa). BSA, Man, and Man-BSA were characterized by ^1^H-NMR with D_2_O as the solvent (Bruker 600 MHz).

### 2.3 Preparation of BSA@Rb1 NPs and Man-BSA@Rb1 NPs

The Man-BSA@Rb1 NPs were obtained by a typical de-solvation method. In brief, Man-BSA (34 mg BSA) was first dissolved in 4.0 ml borate buffer (pH 8.0) and sonicated for 30 min. Rb1 (10 mg) was weighed and dissolved in 1 ml ethanol, and slowly added to the Man-BSA solution to react for 24 h. Man-BSA@Rb1 NPs were obtained by evaporation and centrifugation to remove ethanol and un-encapsulated Rb1. BSA@Rb1 NPs were also prepared in a similar method to Man-BSA@Rb1 NPs. Rb1, BSA@Rb1, and Man-BSA@Rb1 were also characterized by ^1^H-NMR with D_2_O as the solvent (Bruker 600 MHz).

### 2.4 Drug encapsulation efficiency

The drug encapsulation efficiency of Rb1 in BSA@Rb1 NPs and Man-BSA@Rb1 NPs was determined by a typical high-pressure liquid chromatography (HPLC) method. In brief, NPs were dissolved in acetonitrile and sonicated to completely release the drug. The concentration of Rb1 in the supernatant was determined by HPLC. The related conditions included C18 column (4.60 mm × 250 mm, 5 μm), a mobile phase of water/acetonitrile (50%/50%, v/v), the flow rate of 1.0 ml/min, the detection wavelength of 203 nm, and the column temperature of 35°C. The formula for drug encapsulation efficiency was as follows:

The drug encapsulation efficiency=(weight of Rb1 in the NPs)/(weight of total Rb1)×100%

### 2.5 Physicochemical characterization of Man-BSA@Rb1 NPs

The particle size and zeta potential of BSA@Rb1 NPs and Man-BSA@Rb1 NPs were determined by dynamic light scattering (DLS) (Malvern Instruments Ltd., U.K.). The microscopic morphology of Man-BSA@Rb1 NPs was detected using transmission electron microscopy (TEM) (JEM-2100F, JEOL, Japan). The colloidal stability of BSA@Rb1 NPs and Man-BSA@Rb1 NPs was performed by monitoring their particle size in PBS (pH 7.4) for 24 h. The cumulative release of BSA@Rb1 NPs and Man-BSA@Rb1 NPs was tested by incubating them in a dialysis bag (MWCO 3500 Da) with pH 5.0 PBS and pH 7.4 PBS, respectively, and the Rb1 concentration in the medium was determined by HPLC.

### 2.6 Cell toxicity and cellular uptake

The toxicity study of Man-BSA, Rb1, BSA@Rb1 NPs, and Man-BSA@Rb1 NPs was evaluated by a standard CCK8 method in Raw264.7 cells. In brief, the cells were seeded in 96-well plates until ∼80% density and then incubated with different treatments of 10, 25, 50, and 100 μM for 24 h (equal to Rb1 concentration). A standard CCK8 assay was performed to determine the related cytotoxicity.

For the cellular uptake study, Raw264.7 cells were cultured and seeded in confocal dishes for confocal imaging or 12-well plates for flow cytometry measuring. The cells were incubated with BSA@Rb1 NPs and Man-BSA@Rb1 NPs (25 μM, equal to Rb1 concentration) for 4 h, and then stained with DAPI before confocal microscopy or collected before flow cytometry.

### 2.7 Inflammatory cytokines release and protein expression determination

The anti-inflammatory ability of Man-BSA, Rb1, BSA@Rb1 NPs, and Man-BSA@Rb1 NPs was evaluated by kits and the Western blot assay. In brief, Raw264.7 cells were seeded in 6-well plates and pretreated with different treatments (25 μM, equal to Rb1 concentration) for 1 h. After that, the cells were incubated with LPS (1 μg/ml) for another 24 h. The amounts of released cytokines including nitrite, TNF-α, and IL-6 in the medium were assessed by the nitrite assay kit and TNF-α/IL-6 ELISA kits. The cells were washed and isolated according to a standard procedure. The concentrations and levels of p-p65/p65, p-IκB-α/IκB-α, p-JNK/JNK, and p-p38/p38 were determined by a BCA Protein Assay Kit and Western blot, respectively. The intracellular levels of the ROS and calcium Ca^2+^ were determined by ROS assay kit and Fluo-4 AM assay kit, respectively.

### 2.8 Liver accumulation and therapeutic studies

The animals used in this study were approved by the Animal Care and Use Committee of the First Affiliated Hospital of Zhengzhou University. For the liver accumulation study, female C57BL/6 mice (6 weeks old) were randomly divided into two groups of FITC-labeled BSA@Rb1 NPs and Man-BSA@Rb1 NPs (*n* = 3). All mice were set to perform the procedure of intraperitoneal injection of 400 mg/kg d-Gal and 60 μg/kg LPS. After 0.5 h, the above formulations (20 mg/kg Rb1 equivalent) were administrated by intraperitoneal injection. After another 4 h, the mice were sacrificed, the liver was excised, and immunofluorescence staining was performed. For the therapeutic study, female C57BL/6 mice (6 weeks old) were randomly divided into five groups (n = 5) of saline, untreated, Rb1, BSA@Rb1 NPs, and Man-BSA@Rb1 NPs (20 mg/kg Rb1 equivalent). The treatment was performed at 0.5 h before intraperitoneal injection of 400 mg/kg d-Gal and 60 μg/kg LPS. 12 h after intraperitoneal injection, the mice were sacrificed, and the liver and serum were collected for hematoxylin and eosin (HE) staining, and alanine transaminase (ALT), aspartate transaminase (AST), and TNF-α and IL-1β determination, respectively.

### 2.9 Biosafety studies

For a potential toxicity study *in vivo*, female C57BL/6 mice (6 weeks old) were randomly divided into two groups. The treatments were administrated with saline and Man-BSA@Rb1 NPs (20 mg/kg Rb1 equivalent). 12 h after the injection, the mice were sacrificed and the major organs were collected for H&E staining. The blood was collected for alanine aminotransferase (ALT), aspartate aminotransferase (AST) determination, and blood analysis of WBCs, lymphocytes, and neutrophils. For the hemolysis assay, red blood cells (RBCs) were collected and incubated with PBS (pH 7.4), Man-BSA@Rb1 NPs, and 1% Triton. After 2 h, samples were collected and imaged. Then, absorbance of the supernatant was determined with a multimode reader at 540 nm.

### 2.10 Statistical analysis

Statistical analysis was performed by two-sided Student’s t-test for two groups and one-way ANOVA analysis of variance for multiple groups (when *p* < 0.05, the data were considered statistically significant).

## 3 Results and discussion

### 3.1 Preparation and characterization of Man-BSA@Rb1 NPs

**FIGURE 1:**
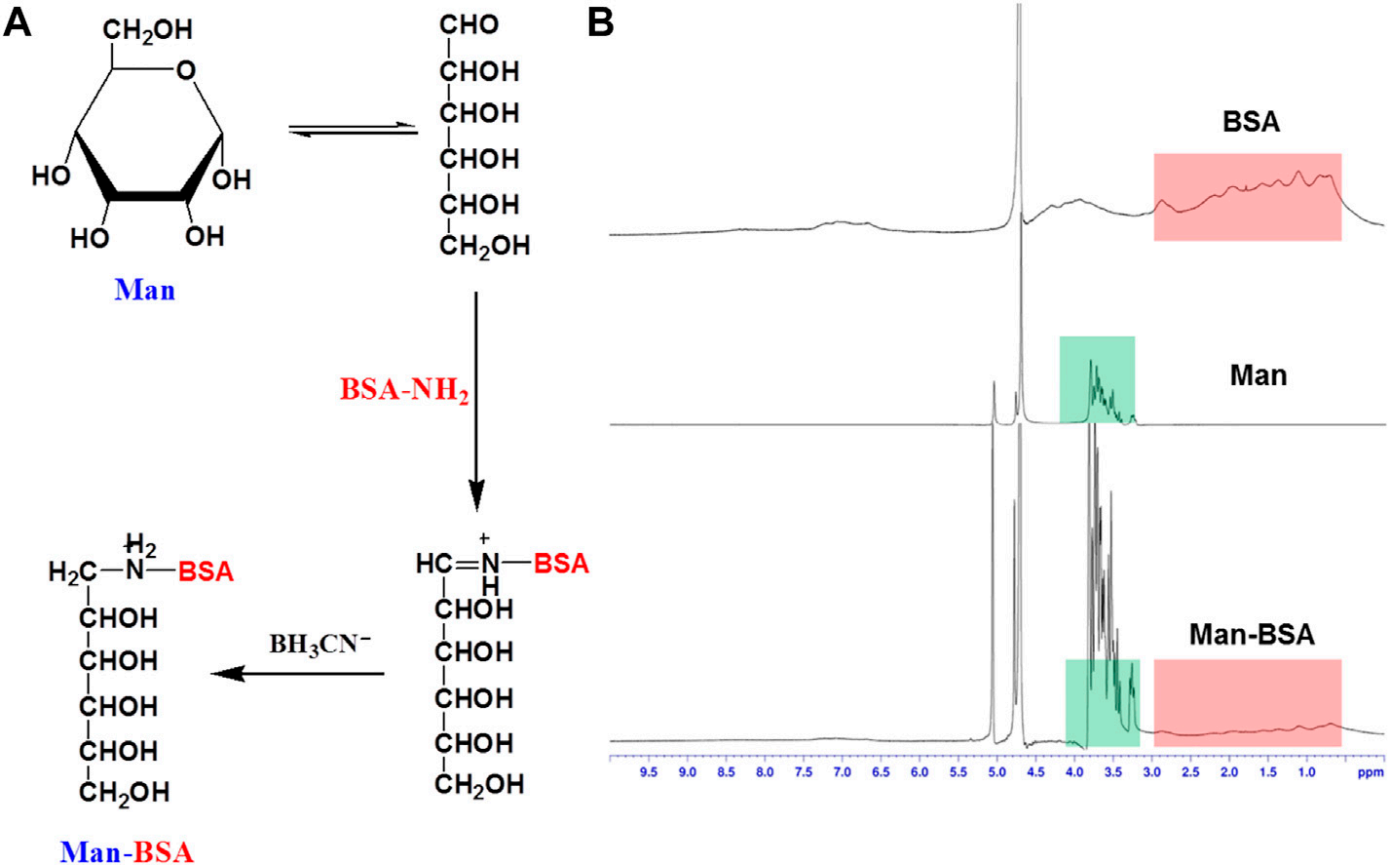
Synthesis and characterization of Man-BSA. **(A)** The synthetic route of Man-BSA. **(B)** The ^1^H-NMR spectra of BSA, Man, and Man-BSA in D_2_O.

**FIGURE 2:**
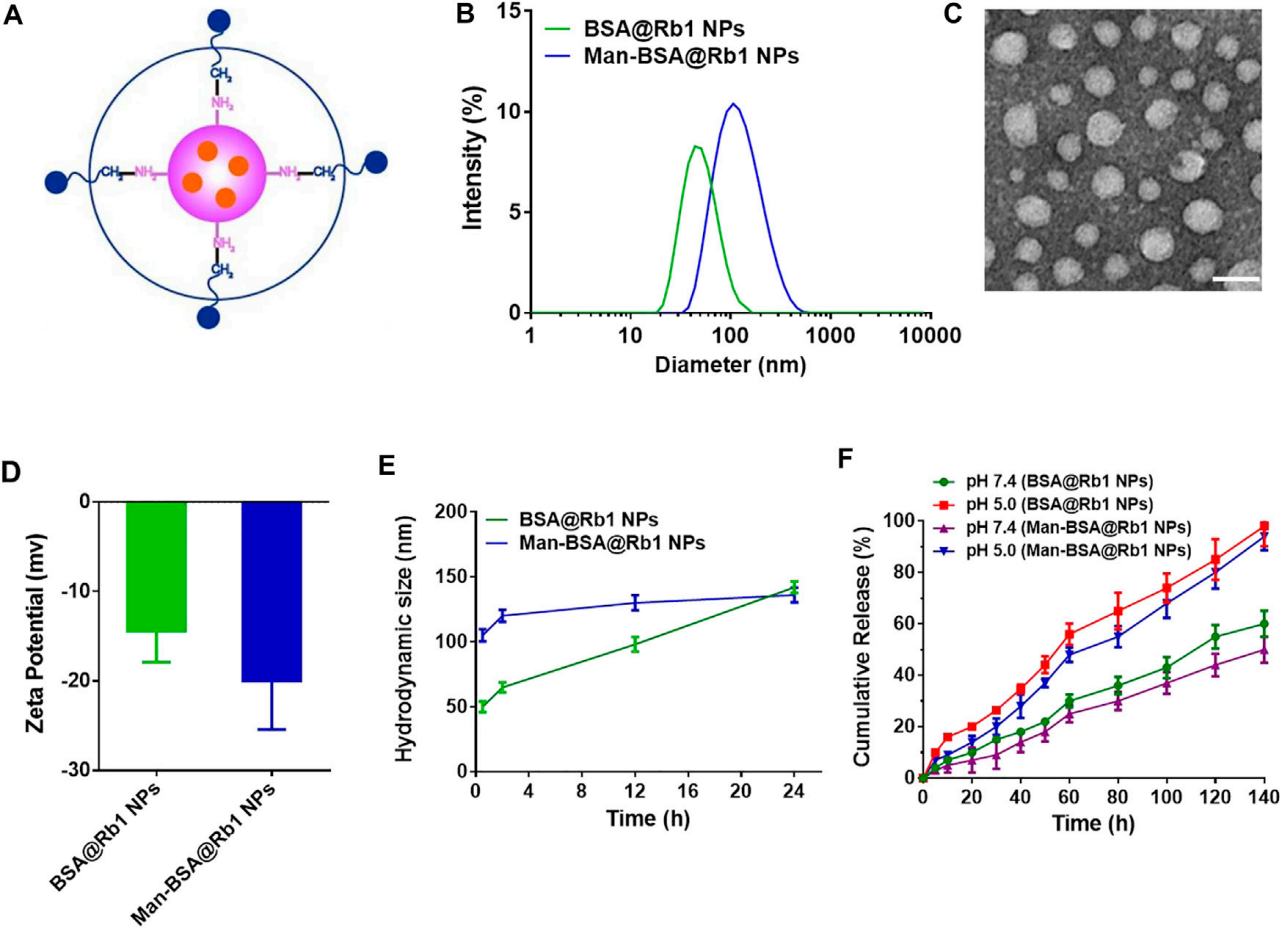
Characterization of Man-BSA@Rb1 NPs. **(A)** Structural diagram of Man-BSA@Rb1 NPs. **(B)** Hydrodynamic size distribution of BSA@Rb1 NPs and Man-BSA@Rb1 NPs. **(C)** Representative TEM images of Man-BSA@Rb1 NPs (scale bar = 100 nm). **(D)** Zeta potential of BSA@Rb1 NPs and Man-BSA@Rb1 NPs. **(E)** Stability of BSA@Rb1 NPs and Man-BSA@Rb1 NPs. **(F)** Cumulative release of BSA@Rb1 NPs and Man-BSA@Rb1 NPs at pH 5.0 and pH 7.4 media.

Man-BSA was first synthesized through the Schiff base reaction between CHO-Man and NH2-BSA (Figure 1A; Mizuta et al., 2021). The structure of Man-BSA was demonstrated by ^1^H-NMR, which was reflected in the characteristic absorption peak of 0.5–3.0 ppm (Man) and 3.2–3.9 ppm (BSA) (Figure 1B). BSA@Rb1 NPs and Man-BSA@Rb1 NPs were then prepared using the de-solvation method (Dong et al., 2019). Their formation was demonstrated by ^1^H-NMR and the size distribution study. The characteristic absorption peak of 3.1–3.4 ppm (Rb1) appeared in BSA@Rb1 NPs, which indicated the feasibility of the preparation method in Man-BSA@Rb1 NPs (Supplementary Figure S1). The average particle size of BSA@Rb1 NPs and Man-BSA@Rb1 NPs was 43 and 106 nm, respectively. The modification of Man on the BSA surface would induce an increase in the particle size of BSA@Rb1 NPs (Figures 2A,B). TEM image confirmed the spherical shape of Man-BSA@Rb1 NPs (Figure 2C). The zeta potential of BSA@Rb1 NPs and Man-BSA@Rb1 NPs was -14.4 mV and −20.0 mV, respectively (Figure 2D). These results indicated the successful preparation of BSA@Rb1 NPs and Man-BSA@Rb1 NPs.

The stability and drug release are essential parameters for the efficacy of encapsulated drugs in NPs (Jana et al., 2020; Zhang H. et al., 2021). Therefore, the stability of BSA@Rb1 NPs and Man-BSA@Rb1 NPs was first evaluated in PBS (pH 7.4) media. As shown in Figure 2E, Man-BSA@Rb1 NPs displayed satisfied stability compared with BSA@Rb1 NPs since no significant size change was observed within 24 h. Then, the stability of Man-BSA@Rb1 NPs was further evaluated by incubating Man-BSA@Rb1 NPs in 10% FBS or a combination of PBS (pH 7.4) and 10% FBS. Results indicated that Man-BSA@Rb1 NPs displayed satisfied stability against different media (Supplementary Figure S2). Next, the release profile of Rb1 in NPs exhibited a pH-dependent manner with an increased release rate at pH 5.0 compared to that at pH 7.4. In addition, the cumulative release percentage of Rb1 in BSA@Rb1 NPs was higher than that in Man-BSA@Rb1 NPs at the same time (Figure 2F). Furthermore, the aqueous solution of Man-BSA@Rb1 NPs was clearer than that of BSA@Rb1 NPs and that of Rb1, indicating the enhanced solubility of Rb1 after encapsulating in NPs and the improved hydrophilicity after Man attachment in BSA. As a result, the encapsulation efficiency of Rb1 in BSA@Rb1 NPs and Man-BSA@Rb1 NPs was 65.3 ± 4.5% and 96.7 ± 6.5%, respectively (Supplementary Figure S3A). These results demonstrated the superior properties of Man-BSA@Rb1 NPs in improved solubility of Rb1.

### 3.2 Cytotoxicity and cellular uptake of Man-BSA@Rb1 NPs

Before the application of Man-BSA@Rb1 NPs in biological functions, we first checked their cytotoxicity in Raw264.7 cells through a CCK8 measurement. As shown in Supplementary Figure S2B, with the increase in concentrations, the carrier of Man-BSA and the drug of Rb1 had no significant effect on the cell viability. However, the cell viability was markedly decreased for Rb1 NPs treatment, especially for Man-BSA@Rb1 NPs (67% viability) when compared with free Rb1 treatment (97% viability) at high concentrations. This result indicated that Rb1 NP formation might increase the cellular uptake of Rb1, leading to enhanced cytotoxicity.

**FIGURE 3:**
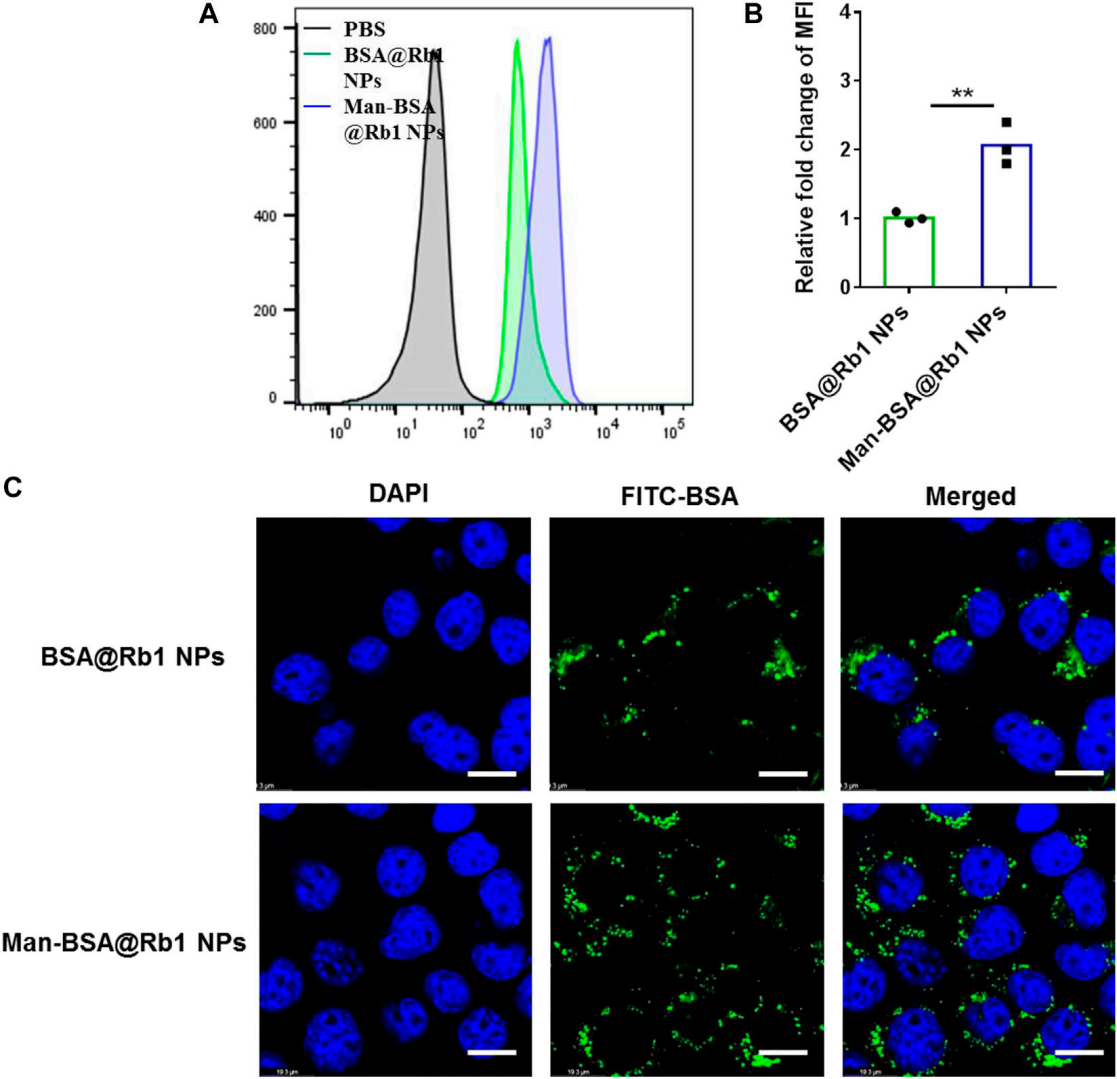
Cellular uptake of FITC-labeled Man-BSA@Rb1 NPs. **(A–B)** Flow histogram and MFI detected by flow cytometry. **(C)** Representative images detected by CLSM. Scale bar = 10 μm.

To evaluate the targeting ability of Man-BSA@Rb1 NPs, the cellular uptake of BSA@Rb1 NPs and Man-BSA@Rb1 NPs was evaluated in Raw264.7 cells by flow cytometry and CLSM. FITC-labeled BSA was used as a tool to quantify the accumulation of NPs in cells. As shown in Figures 3A,B, the mean fluorescence intensity (MFI) of FITC in the Man-BSA@Rb1 NP group was ∼2 folds as high as that of the BSA@Rb1 NPs group. Furthermore, we confirmed these findings by confocal scanning light microscopy (CLSM), as shown in Figure 3C; the green fluorescence intensity showed an increased manner in the Man-BSA@Rb1 NP group compared with the BSA@Rb1 NP group. These results demonstrated that Man modification could facilitate the uptake and endow the targeting ability of Rb1 NPs, which might be due to the overexpressed Man receptor on macrophages.

### 3.3 Anti-inflammatory activity of Man-BSA@Rb1 NPs *in vitro*

**FIGURE 4:**
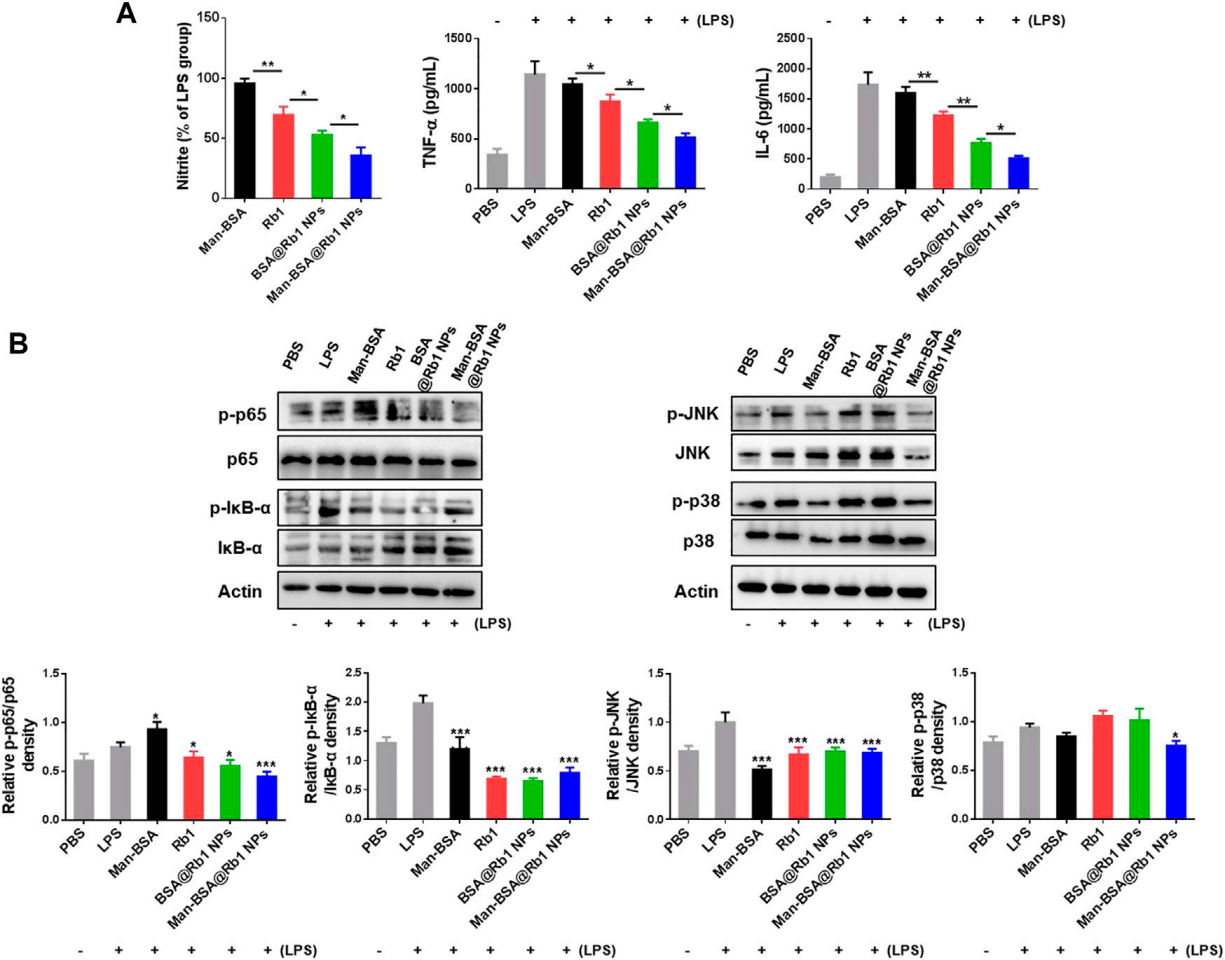
Effects of Man-BSA@Rb1 NPs treatment on LPS-induced inflammatory responses in Raw264.7 cells. **(A)** The levels of nitrite, TNF-α, and IL-6 in the medium of Raw264.7 cells. **(B)** The expression of p-p65/p65, p-IκB-α/IκB-α, p-JNK/JNK, and p-p38/p38 in Raw264.7 cells, and their quantitative analysis by ImageJ.

Some pro-inflammatory cytokines such as nitrite, TNF-α, and IL-6 will be released in the progression of the inflammation occurrence (Yang et al., 2020). To demonstrate the anti-inflammatory activity of Man-BSA@Rb1 NPs, the above mediators were analyzed in LPS-stimulated Raw264.7 cells. As shown in Figure 4A, Man-BSA treatment had no effects on nitrite levels when compared with LPS treatment. Significantly, the main treatments, especially for Man-BSA@Rb1 NP treatment, decreased nitrite levels when compared with Man-BSA treatment. The levels of TNF-α and IL-6 were increased after LPS stimulation, and the main treatments of Rb1, BSA@Rb1 NPs, and Man-BSA@Rb1 NPs significantly decreased their levels when compared with LPS treatment. Similarly, Man-BSA treatment had no effects on levels of TNF-α and IL-6 when compared with LPS treatment.

The signal pathways involved in macrophage activation and inflammatory responses mainly included the NF-κB and MAPK signaling pathways (Li et al., 2021; Smith and Burger, 2021). Therefore, we evaluated the effects of the above treatments on the expression of p65 and IκB-α (key regulators of NF-κB pathway) and JNK and p38 (key regulators of MAPK pathway) proteins. As shown in Figure 4B, LPS stimulation induced a marked increase in the expression of p-p65 and p-IκB-α, suggesting that LPS led to the activation of the NF-κB pathway in Raw264.7 cells. As anticipated, the main treatments could inhibit its activation by decreasing the expression of p-p65 and p-IκB-α. Notably, the treatment of Man-BSA induced an apparent rise in the expression of p-p65. However, the produced Man-BSA@Rb1 NPs achieved the best silence effect. In addition, LPS stimulation activated JNK and p38 phosphorylation. Compared with other treatment groups, Man-BSA@Rb1 NP treatment exhibited the satisfied silence effect in the expression of p-JNK and p-p38. Interestingly, the carrier of Man-BSA had a positive effect in the expression of p-JNK and p-p38. These results demonstrated that Man-BSA@Rb1 NPs could improve the effects of Rb1 on LPS-induced activation of NF-κB and MAPK pathways.

### 3.4 Determination of ROS generation and intracellular calcium (Ca^2+^) levels

**FIGURE 5:**
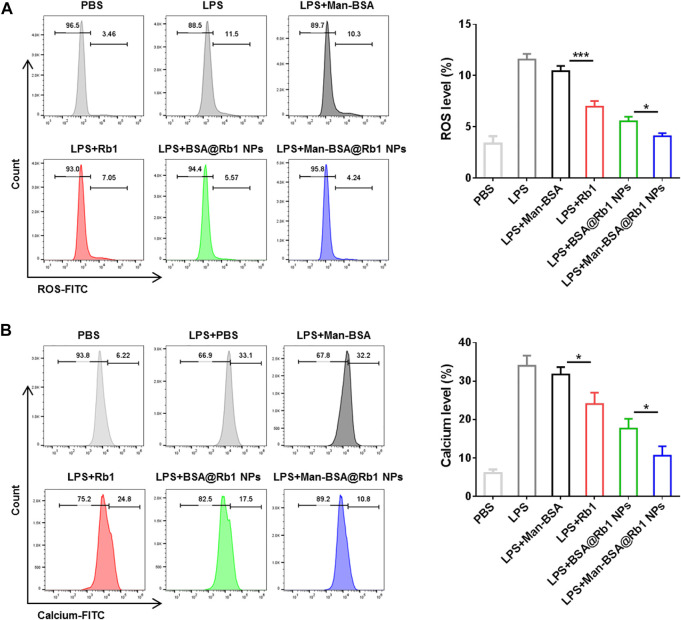
Effects of Man-BSA@Rb1 NP treatment on ROS production and Ca^2+^ levels in Raw264.7 cells. **(A)** The ROS levels in Raw264.7 cells determined by flow cytometry. **(B)** The Ca^2+^ levels in Raw264.7 cells determined by flow cytometry.

ROS generation and Ca^2+^ levels increase are important mediators to reflect the cell damage and inflammation response (Madreiter-Sokolowski et al., 2020). In this study, the effects of Man-BSA@Rb1 NPs on the levels of ROS and Ca^2+^ were evaluated in LPS-induced Raw264.7 cells. As shown in Figure 5, after LPS stimulation, the levels of ROS and Ca^2+^ were significantly increased when compared with the PBS group. The treatments of Rb1 and BSA@Rb1 NPs could effectively decrease the levels of ROS and Ca^2+^, while Man-BSA@Rb1 NPs exhibited the best outcome. Interestingly, the delivery carrier of Man-BSA had no effects on the levels of ROS and Ca^2+^. Therefore, Man-BSA might be a potential carrier to improve the anti-inflammatory activity of Rb1, which was also demonstrated in the aforementioned results.

### 3.5 Liver accumulation and therapeutic efficacy of Man-BSA@Rb1 NPs *in vivo*

**FIGURE 6:**
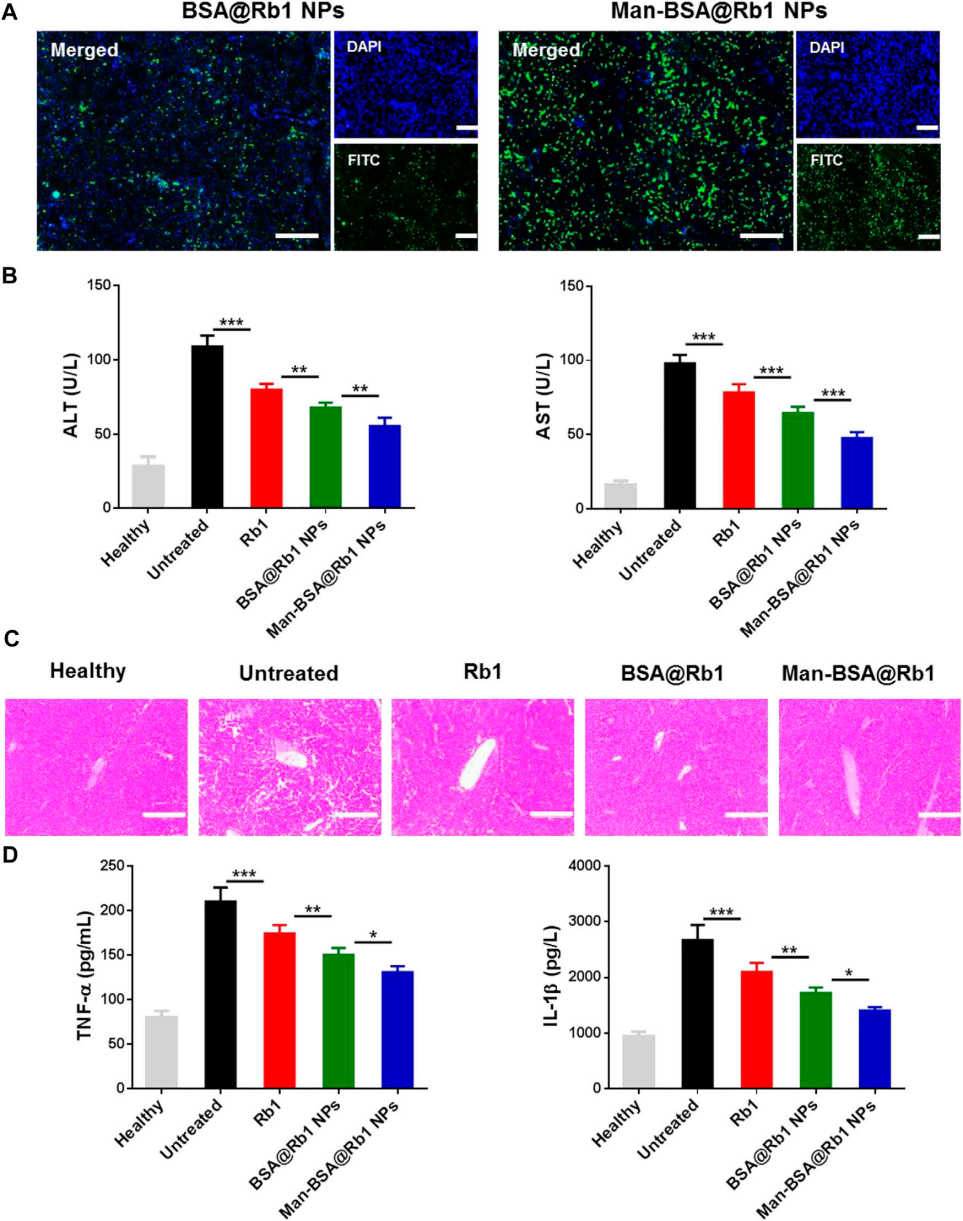
Biodistribution and therapeutic efficacy of Man-BSA@Rb1 NPs in d-Gal/LPS-induced liver injury model. **(A)** Analysis of liver accumulation of NPs in liver (scale bar = 200 μm). **(B)** Levels of ALT and AST in serum. **(C)** Representative images of H&E staining of liver tissue sections (scale bar = 200 μm). **(D)** Levels of TNF-α and IL-1β in serum.

After demonstrating the targeting ability of Man-BSA@Rb1 NPs *in vitro*, their targeting ability was next evaluated by detecting the liver accumulation in injured liver using BSA@Rb1 NPs as controls. As shown in Figure 6A, a strong fluorescent signal was observed in the Man-BSA@Rb1 NP group, while the BSA@Rb1 NP group exhibited the weakest fluorescent signal. Therefore, Man-BSA@Rb1 NP treatment was expected to achieve the best therapeutic efficacy due to the increased Rb1 delivery to the liver.

Next, the anti-inflammatory activity of Man-BSA@Rb1 NPs was evaluated in Gal/LPS-induced liver injury model. The related therapeutic efficacy was assessed by detecting serum levels of ALT and AST (key biomarkers of liver function), analyzing H&E staining of liver sections (histological analysis of liver morphology), and testing the change in TNF-α and IL-1β levels (key mediators of pro-inflammatory factor). As shown in Figure 6B, ALT and AST levels were significantly increased in the untreated group compared with the healthy group. After different treatments, especially for Man-BSA@Rb1 NPs, ALT and AST levels were decreased compared with the untreated group. In Figures 6C, D, H&E results also revealed that the liver structure tended to be normal when compared with the untreated group. Alterations in the pattern of TNF-α and IL-1β levels resembled the trend of ALT and AST levels.

### 3.6 The safety evaluation of Man-BSA@Rb1 NPs *in vivo*

The potential toxicities of the adopted carrier are ignored factors for the application of the loaded drug. Therefore, the *in vivo* safety of Man-BSA@Rb1 NPs was evaluated by monitoring the liver function, histological section of major organs, and levels of inflammatory cells (Supplementary Figure S3). The results showed that there was no significant change in the levels of ALT and AST between the saline group and Man-BSA@Rb1 NPs. In addition, no pathological change was found in the Man-BSA@Rb1 NP group compared with the saline group, indicating the good biocompatibility of Man-BSA@Rb1 NPs in major organs. The blood analysis of white blood cells (WBCs), lymphocytes, and neutrophils were also performed, and results showed that there was no notable change in the number of blood cells between the Man-BSA@Rb1 NP group and the saline group. Moreover, the hemolytic activity of Man-BSA@Rb1 NPs was evaluated, and results showed that Man-BSA@Rb1 NP treatment exhibited the negligible level of hemolysis.

## 4 Conclusion

In summary, we have engineered a novel delivery carrier of Man-BSA, which can simultaneously increase Rb1 solubility and endow Rb1 NPs with targeting ability. The fabricated Man-BSA@Rb1 NPs showed attractive properties with a uniform size distribution, spherical shape, high encapsulation efficacy, satisfying stability, and pH-dependent release. The cellular uptake and liver distribution studies demonstrated that Man modification could increase the accumulation of BSA@Rb1 NPs in cells and liver. The improved anti-inflammatory efficacy of Man-BSA@Rb1 NPs was demonstrated in LPS-induced Raw264.7 cells *in vitro* and d-Gal/LPS-induced liver injury model *in vivo*. The related molecular mechanisms were potently involved by inhibiting NF-κB and MAPK signaling pathways, including p-p65/p65, p-IκB-α/IκB-α, p-JNK/JNK, and p-p38/p38 proteins. In addition, Man-BSA@Rb1 NPs exhibited acceptable biosafety *in vivo*. The results presented in this study demonstrate that Man-BSA can play the ideal carrier for Rb1 delivery, which has the potential as an effective delivery platform for enhanced anti-inflammatory therapy.

## Data Availability

The raw data supporting the conclusion of this article will be made available by the authors, without undue reservation.
