# Supplementary material for: Mannose-decorated ginsenoside Rb1 albumin nanoparticles for targeted anti-inflammatory therapy
Source: Front Bioeng Biotechnol. 2022 Aug 15;10:962380. doi: 10.3389/fbioe.2022.962380 (PMC9420840; doi:10.3389/fbioe.2022.962380)
Supplement: Supplementary file 1 [file DataSheet1.docx]

**Mannose-decorated Ginsenoside Rb1 Albumin Nanoparticles for Targeted anti-inflammatory therapy**

Zhihui Fu^1#^, Xiaohui Wang^1#^, Xuan Lu^2^, Ying Yang^2^, Lingling Zhao^1^, Lin Zhou^1^, Kaikai Wang^2*^, Hanlin Fu^1*^

1. The First Affiliated Hospital of Zhengzhou University, Zhengzhou City, Henan Province, China.

2. School of Pharmacy and Affiliated Hospital of Nantong University, Nantong University, Nantong, Jiangsu Province, China.

#Contributed equally

*Correspondence to: kirk2008@126.com, fuhanlinzzdx@163.com.


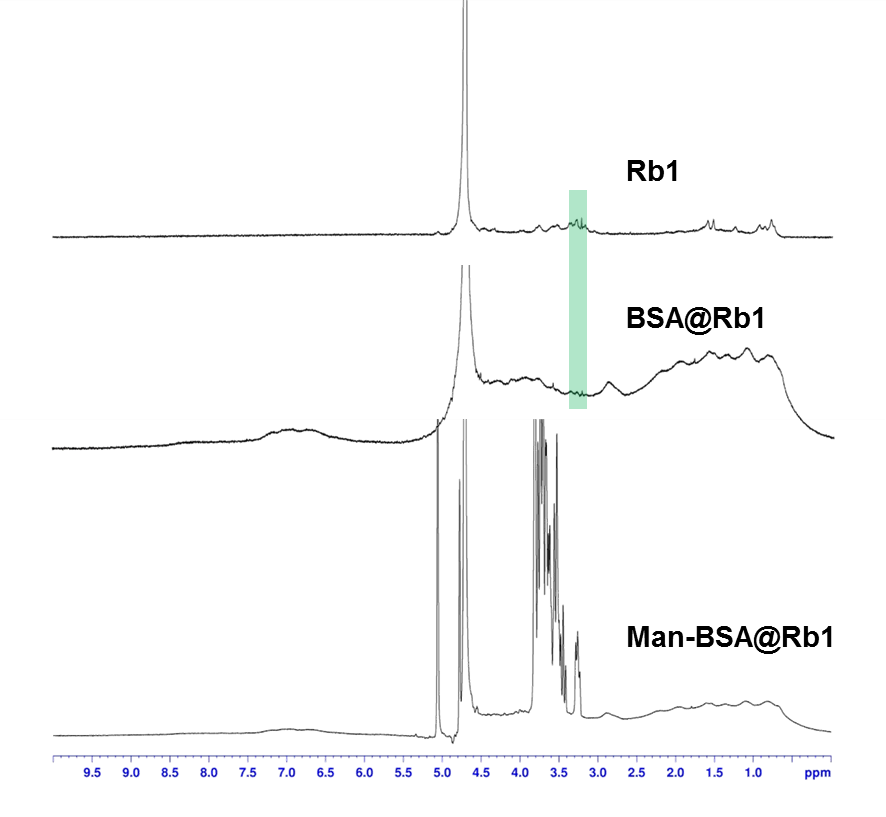


**Figure S1.** The ^1^H-NMR spectrums of Rb1, BSA@Rb1 NPs and Man-BSA@Rb1 NPs in D_2_O.


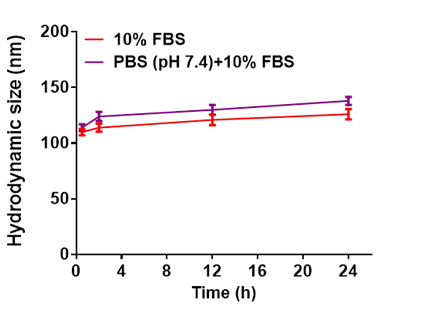


**Figure S2.** Stability of Man-BSA@Rb1 NPs in 10% FBS or a combination of PBS (pH 7.4) and 10% FBS.


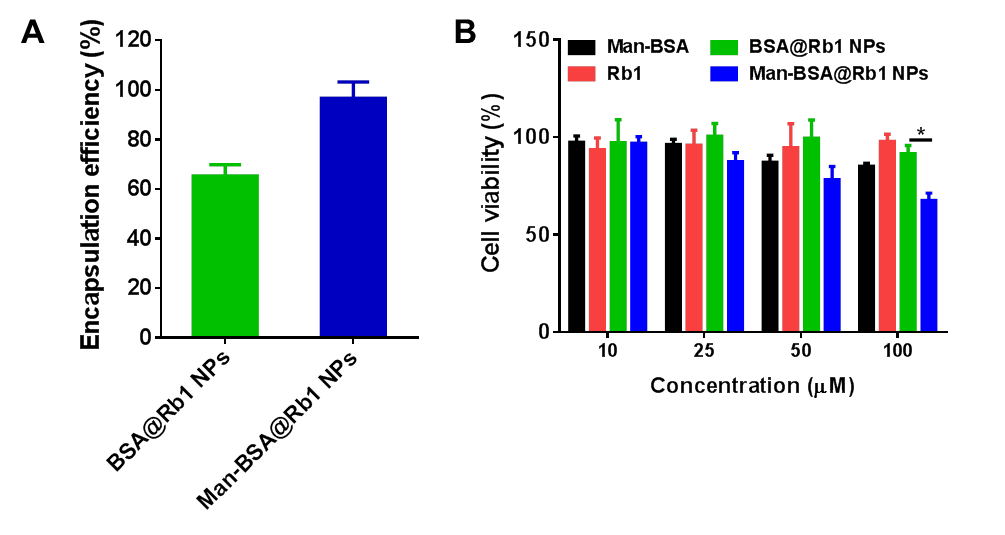


**Figure S3.** (A) The encapsulation efficiency of Rb1 in BSA@Rb1 NPs and Man-BSA@Rb1 NPs. (B) The cytotoxicity of Man-BSA, Rb1, BSA@Rb1 NPs and Man-BSA@Rb1 NPs in Raw264.7 cells.


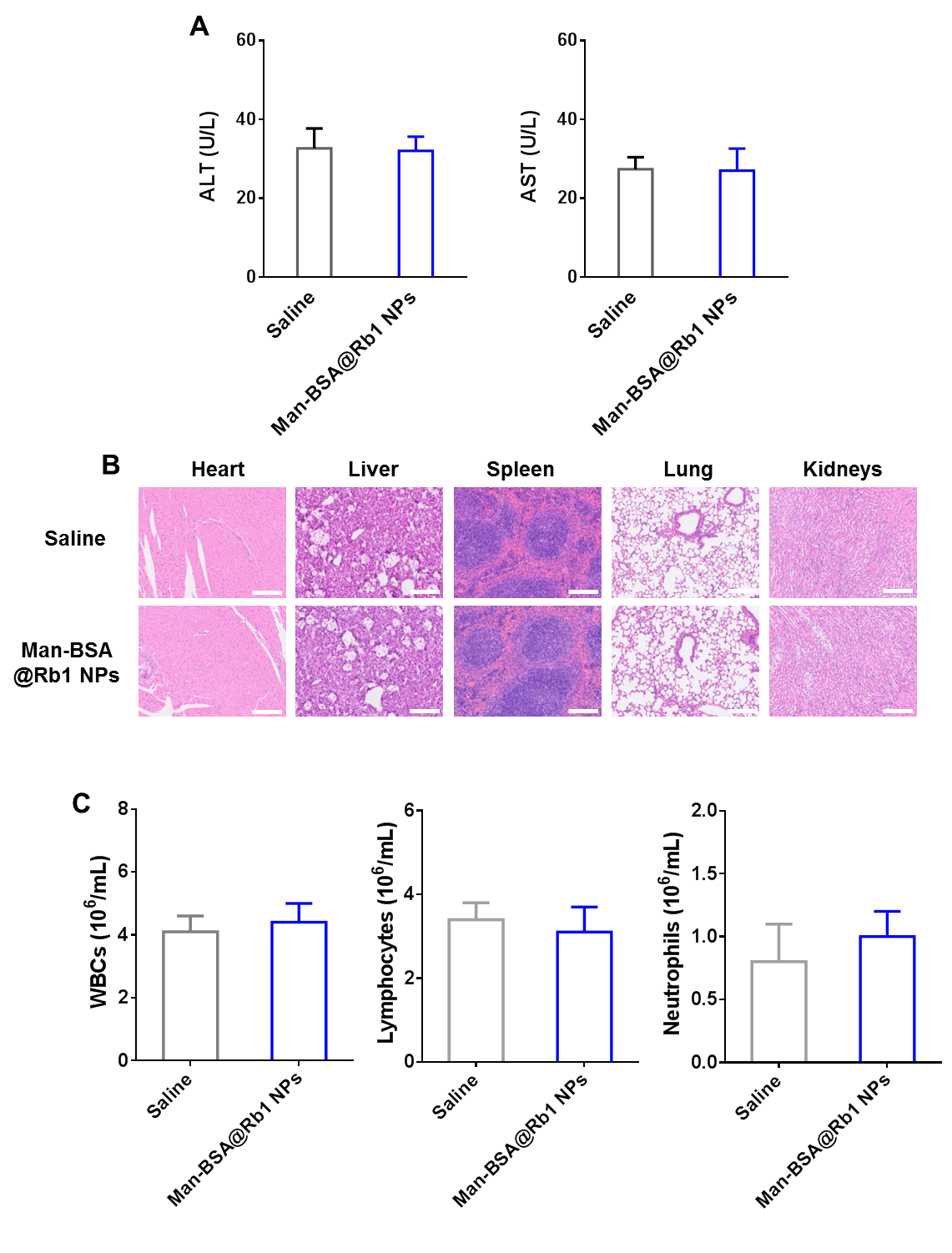


**Figure S4**. (A) Levels of ALT and AST in serum. (B) Representative of H&E staining of major organs. (C) Levels of white blood cells (WBCs), lymphocytes and neutrophils in serum. Scale bar = 200 μm.


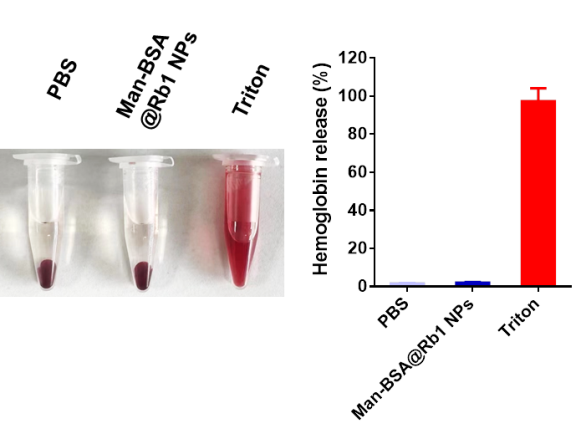


**Figure S5.** The hemolytic activity of Man-BSA@Rb1 NPs on RBCs.
